# Deep Neural Networks Outperform the CAPRA Score in Predicting Biochemical Recurrence After Prostatectomy

**DOI:** 10.3389/fonc.2020.607923

**Published:** 2021-02-11

**Authors:** Paul Sargos, Nicolas Leduc, Nicolas Giraud, Giorgio Gandaglia, Mathieu Roumiguié, Guillaume Ploussard, Francois Rozet, Michel Soulié, Romain Mathieu, Pierre Mongiat Artus, Tamim Niazi, Vincent Vinh-Hung, Jean-Baptiste Beauval

**Affiliations:** ^1^ Department of Radiation Oncology, Institut Bergonié, Bordeaux, France; ^2^ Division of Radiation Oncology, Department of Oncology, McGill University, Montreal, QC, Canada; ^3^ Division of Oncology, Unit of Urology, Urological Research Institute, IRCCS Ospedale San Raffaele, Milan, Italy; ^4^ Department of Urology, CHU de Toulouse, Toulouse, France; ^5^ Department of Urology, Clinique La Croix du Sud, Quint-Fonsegrives, France; ^6^ Department of Urology, Institut Mutualiste Montsouris, Paris, France; ^7^ Department of Urology, CHU de Rennes, Rennes, France; ^8^ Department of Urology, Hôpital Saint Louis, Paris, France; ^9^ Department of Radiation Oncology, Hôpital Clarac, CHU de la Martinique, Fort-de-France, France

**Keywords:** prostate cancer, machine learning, predictive, recurrence, biochemical

## Abstract

**Background:**

Use of predictive models for the prediction of biochemical recurrence (BCR) is gaining attention for prostate cancer (PCa). Specifically, BCR occurs in approximately 20–40% of patients five years after radical prostatectomy (RP) and the ability to predict BCR may help clinicians to make better treatment decisions. We aim to investigate the accuracy of CAPRA score compared to others models in predicting the 3-year BCR of PCa patients.

**Material and Methods:**

A total of 5043 men who underwent RP were analyzed retrospectively. The accuracy of CAPRA score, Cox regression analysis, logistic regression, K-nearest neighbor (KNN), random forest (RF) and a densely connected feed-forward neural network (DNN) classifier were compared in terms of 3-year BCR predictive value. The area under the receiver operating characteristic curve was mainly used to assess the performance of the predictive models in predicting the 3 years BCR of PCa patients. Pre-operative data such as PSA level, Gleason grade, and T stage were included in the multivariate analysis. To measure potential improvements to the model performance due to additional data, each model was trained once more with an additional set of post-operative surgical data from definitive pathology.

**Results:**

Using the CAPRA score variables, DNN predictive model showed the highest AUC value of 0.7 comparing to the CAPRA score, logistic regression, KNN, RF, and cox regression with 0.63, 0.63, 0.55, 0.64, and 0.64, respectively. After including the post-operative variables to the model, the AUC values based on KNN, RF, and cox regression and DNN were improved to 0.77, 0.74, 0.75, and 0.84, respectively.

**Conclusions:**

Our results showed that the DNN has the potential to predict the 3-year BCR and outperformed the CAPRA score and other predictive models.

## Introduction

Radical prostatectomy (RP) with a concomitant pelvic lymph node dissection is one of the standard treatment for patients with intermediate-risk prostate cancer (PCa) according to the D’Amico classification (1). However, in this population, extremely heterogeneous definitions and outcomes have been reported, and more precise stratification is desirable to guide decision making (2, 3). In this context, the CAncer of the Prostate Risk Assessment (CAPRA) score was developed in 2005 with a patient population from the Cancer of the Prostate Strategic Urologic Research Endeavor (CaPSURE) cohort, which included 1,439 men who had undergone RP, followed in a longitudinal, community based disease registry of patients with prostate cancer (4). The CAPRA score is a pre-treatment scoring system which substratifies patients into 8 risk categories according to five variables from clinical, biochemical and histopathological data. The CAPRA score was built in order to further assess the risk of biochemical and metastatic recurrence among patients treated with RP (5). The same team similarly has been developed a post-operative score, the CAPRA-S score, with improved accuracy *via* incorporation of pathologic data from the RP specimen (6). Noted that the CAPRA score technique outperforms the limitations of counterparts such as D’Amico classification or national comprehensive cancer network (NCCN) score, at predicting several endpoints (7). We note also that the risk nomograms offer more precise risk stratification and prediction, while the calculations can be cumbersome (7). Consequently, an automatic tool based on machine learning (ML) algorithms is needed to predict outcomes following RP, and to guide adjuvant or salvage treatment.

ML algorithms like logistic regression and Cox proportional hazard regression have been employed in the healthcare statistics field for several decades (8, 9). Specifically, logistic regression uses a logit transform to provide event probabilities from input variables, while Cox regression considers the risk of an event occurring based on a linear combination of the covariates. We note that the ML models (e.g., random forest, nearest neighbors) cannot applied directly in predicting the survival outcomes since they don’t consider the censored data (10). To solve this issue some imputation techniques could be considered, like to use the imputation of survival time with random forest model to predict the survival (11). Recently, deep neural networks algorithms have shown promising results in medical applications (12) in order to improve the diagnostic accuracy (13, 14) For example, The Memorial Sloan Kettering Cancer Center (MSKCC) in the United Statesoffers a tool, probably less frequently used in Europe than the CAPRA score, to predict the probability of 2-, 5-, 7-, 10-, and 15-year BCR-free survival after prostate cancer surgery. This tool considers the predictive models like linear regression, logistical regression, and survival progress models to show the cancer recurrence prediction (15, 16).

In this study, using data from a multicentric national database, we aim to compare the accuracy of CAPRA score and others models to predict biochemical recurrence (BCR)-free survival for patients treated with RP. Also, we aim to consider the ML algorithms using the pathological data that considered in CAPRA-S score to improve the accuracy of the predictive models.

## Methods

### Patients

A total of 5,043 patients who underwent RP between 2000 and 2015 for clinically localized prostate carcinoma in six French university hospitals were analyzed retrospectively. All patients underwent a multicore transrectal ultrasound-guided prostate biopsy after digital rectal examination. The Gleason score and percentage of involved biopsies were assigned by dedicated pathologists. Pretreatment PSA was recorded in all men. The clinical stage was assigned by the attending urologist according to the American Joint Committee on Cancer TNM guidelines in effect at the time of inclusion. All patients were preoperatively staged for metastases with a contrast-enhanced abdominal and pelvic computed tomography (CT) and bone scan. The patients received no neoadjuvant/adjuvant hormone therapy or radiation therapy. The CAPRA score was calculated from the available pretreatment variables, and the patients were grouped according to the resulting CAPRA score for analysis (5). Biochemical recurrence after RP was defined according to the American Urological Association (AUA) guidelines as two consecutive PSA values ≥ 0.2 ng/mL at any time post-operatively or any additional treatment more than 6 months after RP (17). The analysis was restricted to patients with a follow-up duration of longer than 12 months.

### Statistical Analysis

The 3-year BCR probability from the CAPRA score assigned for each patient in our cohort was compared to the original CAPRA score related 3-year BCR from the original CaPSURE cohort, using a Kaplan Meier survival analyses. The 3-year BCR probability corresponding to the CAPRA score from the original CaPSURE cohort was assigned to each patient and compared to the actual BCR outcome at 3 years. Non recurring patients who were lost prior to follow-up before 3 years were handled by inferring the survival probability though Kaplan-Meyer actuarial estimation according to the split-and-weighting methods described in Zupan et al. (10). Then, a multivariate predictive model using Cox regression under the assumption of proportional hazards was performed using the variables required for CAPRA score computation (pre-operative PSA, Gleason score and T stage).

### Machine Learning Algorithms and Models Definitions

The results were compared to predictions of BCR by a set of ML models. We performed a binary classification using KNN, RF, logistic regression and DNN. We note that the DNN sequential architecture comprised several fully connected layers that included a varying number of nodes. An input layer takes numeric and one-hot encoded categorical variables and propagates information through the layers. The last layer comprises a single node that outputs the three-year BCR as a single-class probability. All the details of the considered ML models are reported in **Supplementary Material**. We considered the single split, where we divided sample randomly into training (80%) and testing (20%) set, train the classifier models using the training sample and test the models using the test samples. The outcome classes in the training set were weighed to compensate for the initial imbalance in survival status. To achieve the trade-off metrics on the test subset, we tuned the hyperparameters on the training subset using a step-by-step grid search. Area under the curve (AUC) of the receiver operating characteristics (ROC) was measured to assess the performance of the predictive models in predicting 3-year BCR on the test set.

To measure potential improvements to the model performance due to additional data, each model was trained once more with an additional set of post-operative surgical data from definite pathology. Note that the available post-operative variables were not sufficient to compute the CAPRA-S score. For this reason, we combined the pathological tumor stage (pT), pathological lymph nodes dissection status (pN), margin status, prostate volume and surgical Gleason score and used them as input to the predictive models. The performance on the test set was compared with previous results.

The scikit-learn 0.21.2 implementation for Python v3.7.4 was used to run the conventional ML models. The Cox proportional hazard model was computed with the Lifelines v0.22.2 implementation for Python v3.7.4 and double-checked with JMP10.0 (SAS Institute Inc., Cary, NC). We used the Keras v2.2.4 frontend for TensorFlow v1.14 (18) to develop the neural network model. The TensorBoard callback library was used for visualization of the results and optimization. For each hyperparameter, the range and step used in the grid search, over numeric parameters, as recommended, are summarized in the **Supplementary Material**.

## Results

### Patient Characteristics

Among 5,043 patients, 803 cases were excluded due to missing clinical (n=83), biochemical (n=9), pathological (n=338) or follow-up (n=98) data; 275 patients underwent subsequent adjuvant therapy and were ultimately excluded. Thus, the complete records of 4246 patients were available for analysis, as reported in **Table 1**. The characteristics of our cohort were compared to those of the CaPSURE cohort, which was initially used to build the CAPRA score. Results including all variables used in our data set and in the CaPSURE cohort are presented in **Table 2**. Repartition of the CAPRA scores from our cohort and CaPSURE cohort are summarized in **Table 3**. The median CAPRA score of our cohort was 3, compared to 2 for the CaPSURE cohort. The median follow-up duration was 49 months, while the minimum follow-up duration was 12 months. Overall, biochemical recurrence occurred in 817 (19%) of the patients in our cohort with a median of 25 months after RP, compared to 15% with a median of 22 months in the CaPSURE cohort.

**Table 1:** Patient characteristics: pre- and post-operative clinical and pathological variables from 4246 patients included in the analysis.

Pre-operative variables		
Features	Median (min-max)	Mean (95% CI)
Age (years)	67(41–92)	67 (+/− 7, 6)
PSA (ng/mL)	8.7(2.1–44)	9.4 +/− 4
Number of biopsies	3(2–49)	4
Involved biopsies	3(1–21)	4.0 +/− 3
Average involvement of biopsy (%)	20 (0.2–100)	18
Categorical data		Count
CT	T1	2,655
	T2	1,581
	T3	10
Grade Group on biopsy	1	1,101
	2	2,153
	3	834
	Other	169
**Post-operative variables**		
Categorical data		Count
Lymph node dissection	Yes	1,984
	No	2,099
	Missing data	163
Gleason grade group	1	665
	2	1,623
	3	1,498
	4	200
	5	81
	Missing data	178
pT	pT2	2,510
	pT3a	1,360
	pT3b	355
	Missing data	12
	pT4	8
Margin	R0	3,203
	R1	1,039
	Missing data	4
Prostate volume	47 (8-220)	51 +/− 22

**Table 2:** Patient’s variables repartition between our current dataset and the CaPSURE cohort with the corresponding CAPRA point attribution.

Variable	CAPRA point	Current dataset	CaPSURE cohort
**PSA (ng/mL)**			
2.1–6	0	1,112 (26)	721 (50.1)
6.1–10	1	1,392 (32)	453 (31.5)
10.1–20	2	1,734 (40)	209 (14.5)
20.1–30	3	6 (0)	36 (2.5)
>30	4	2 (0)	20 (1.4)
**Biopsy Gleason score (primary/secondary)**			
(1–3)/(1–3)	0	1,218 (29)	1,068 (74.2)
(1–3)/(4–5)	1	2,160 (51)	239 (16.6)
(4–5)/(1–5)	3	868 (20)	132 (9.2)
Clinical T score			
T2-T2	0	4,236 (100)	1,410 (98)
T3a	1	10 (0)	29 (2)
**Percentage of positive biopsies**			
<34	01	2,214 (52)	911 (63.3)
>34		2,032 (48)	528 (36.7)
**Age (years)**			
<50	0	35 (0)	51 (3.5)
>50	1	4,211 (100)	1,388 (96.5)

**Table 3:** Repartition of the patient’s CAPRA scores from our cohort and the CaPSURE cohort are cohort.

CAPRA score	Our cohortNumber of patients (%)	CaPSURE cohortNumber of patients (%)
0	45 (1)	18 (1.3)
1	464 (10)	383 (26.6)
2	1,566 (37)	432 (30)
3	1,153 (27)	293 (20.6)
4	585 (13)	155 (10.8)
5	274 (6)	84 (5.8)
6	151 (3)	43 (3)
7	8 (0)	21 (1.5)

### CAPRA Score and Multivariate Analysis

**Figure 1:**
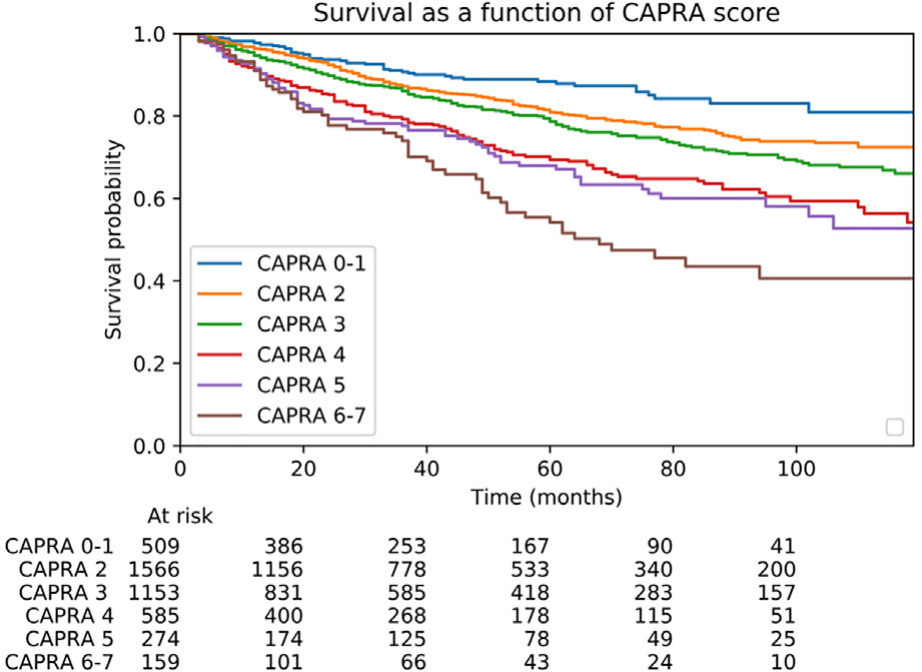
BCR-free survival probability according to the CAPRA score.

Patients with CAPRA scores of 2 and 3 accounted for 64% of the population in our cohort (**Table 2**). Patient survival according to the CAPRA score is shown in **Figure 1**. Regarding the performance of the CAPRA score for predicting biochemical recurrence at 3 years, the c-index was 0.63. Similarly, Cox regression analysis using the same variables (age, Gleason score, involved biopsy percentage, clinical tumor stage, and PSA) predicted recurrence with a c-index of 0.64.

**Figure 2:**
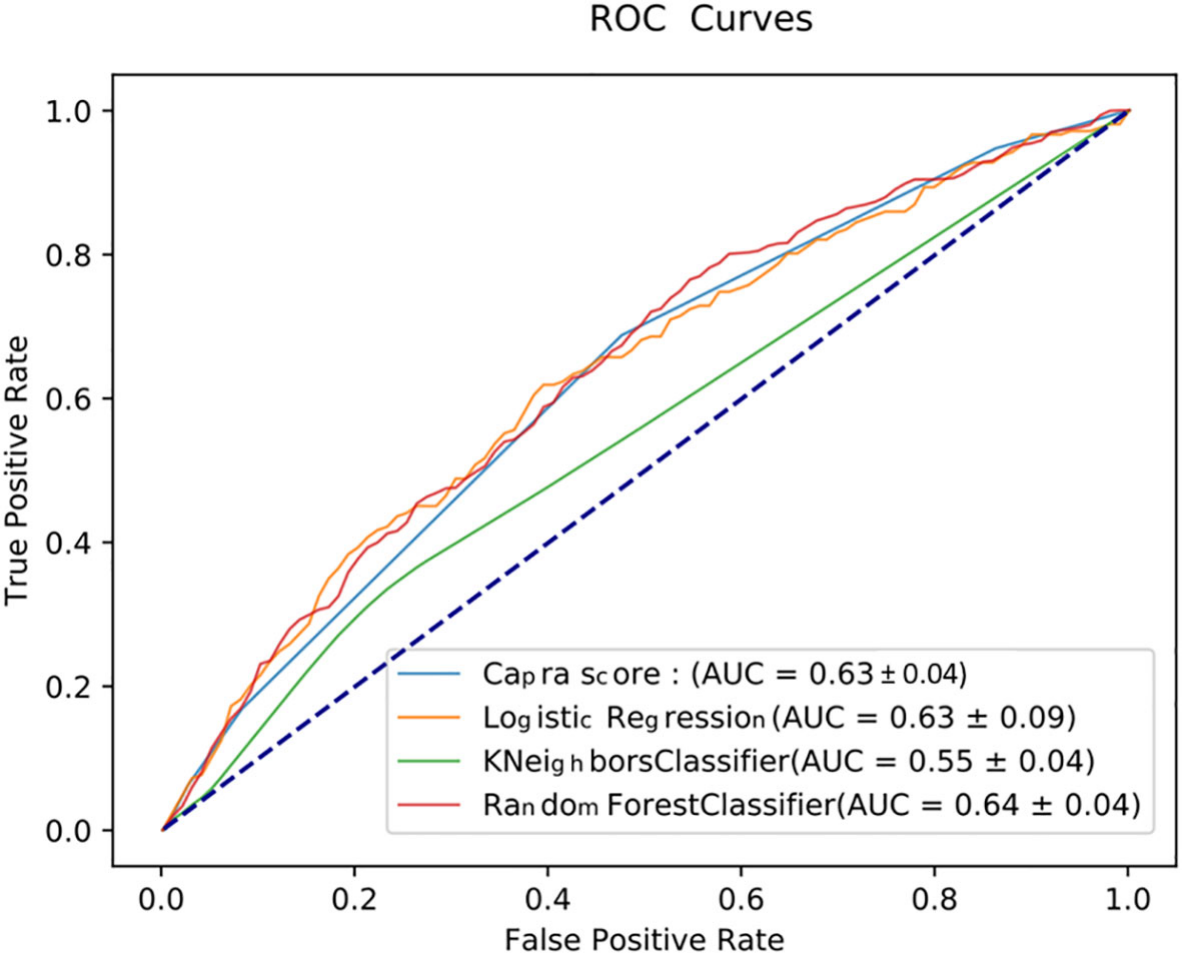
ROC curves measuring the performance of various predictive models in predicting the 3-year BCR using the five input variables of the CAPRA score.

**Figure 2** illustrates the AUC- ROC for the predictive models when the input features are restricted to CAPRA score variables. Considering these pre-operative variables, we found that the DNN model is given the highest AUC value of 0.7 compared to the CAPRA score, logistic regression, KNN, RF, and cox regression with AUC value of 0.63, 0.63, 0.55, 0.64, and 0.64, respectively.

**Figure 3:**
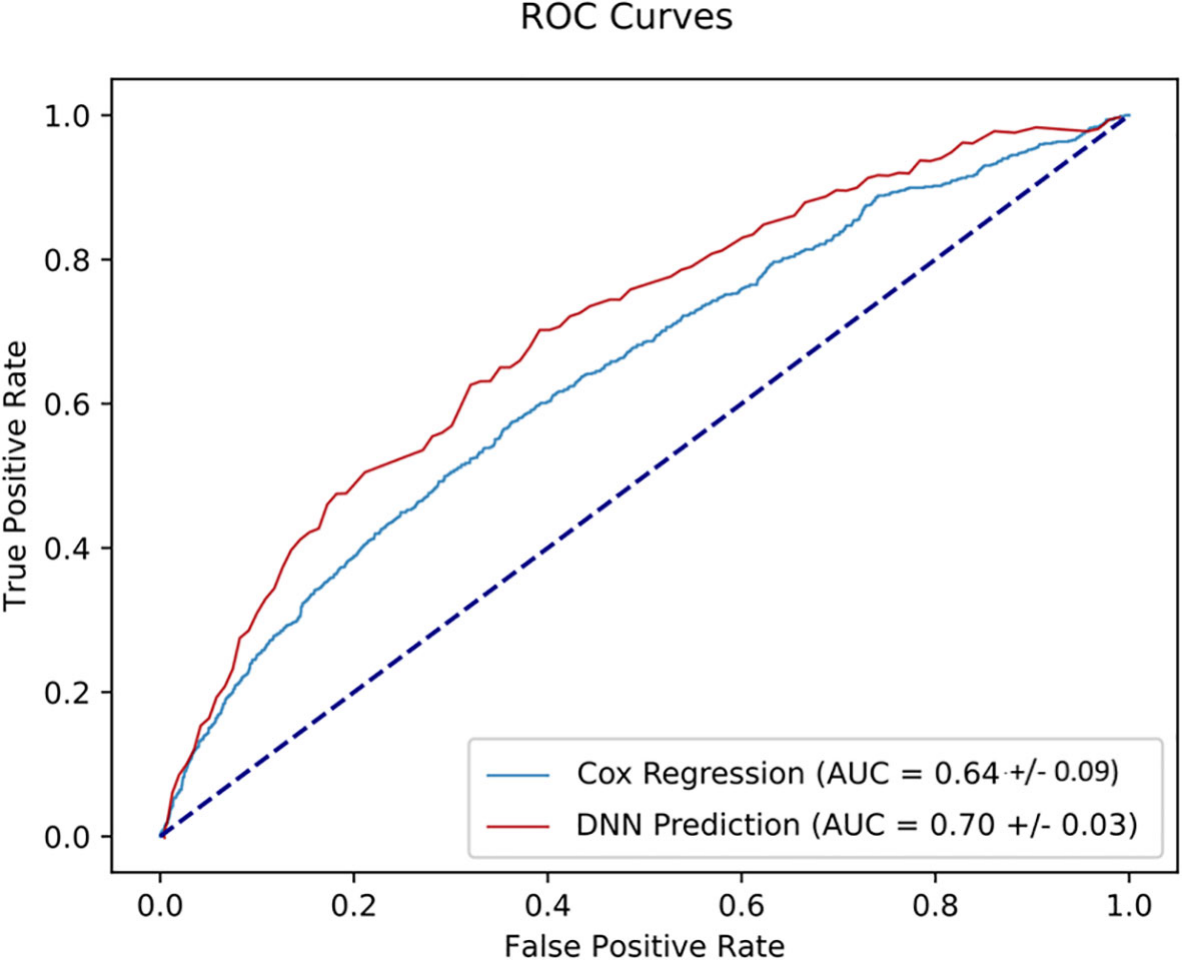
ROC curves measuring the performance of various predictive models in predicting the 3-year BCR using the five input variables of the CAPRA score and post-operative variables.

One more time, we found that the DNN model shows the highest AUC value of 0.84 compared to logistic regression, KNN, RF, and cox regression with AUC value of 0.77, 0.58, 0.74 and 0.75, respectively, using the combined pre- and post- operative variables (pT, pN, margin status, prostate volume and surgical Gleason score) (**Figure 3**).

## Discussion

In this retrospective multi-institutional study, we investigated and compared the potential of CAPRA score and predictive models in predicting the BCR risk after RP using routine variables. We note that the CAPRA score is a commonly used prediction model for the occurrence of biochemical and clinical recurrences developed from the CaPSURE registry (5) with many studies providing external validation with other cohorts (7, 19). In this study, we found that CAPRA score showed a c-index of 0.63 to predict the 3-year-BCR rates with the prognostic variables obviously differing from those of the original CaPSURE cohort.

Overall, the median CAPRA score of our patient cohort was higher, compared to the CAPRA score from the CaPSURE cohort, suggesting a worse prognosis in our series. However, our cohort revealed better survivals among our patients. Other factors may further limit the performance of the CAPRA score: despite substratification of our cohort according to the CAPRA score, most patients (64%) remained in CAPRA score groups 2 and 3, thus reducing the discriminatory power of the score. The heterogeneous nature and prognosis of this intermediary-risk population (2) are not accurately captured by the D’Amico classification and CAPRA score, thus reducing the c-index. Interestingly, while the original study reported a c-index of 0.66 for this score, almost all validation studies published thereafter have reported much higher c-indexes, up to 0.81, raising concerns of bias (20).

With the same restricted set of 5 input variables, predictive models have been able to provide more accurate predictions on a test set after training and tuning the hyperparameters. Specifically, a DNN model showcased the best performance metrics compared to logistic regression, KNN, RF, and cox regression. Our findings are consistent with many published data. For example, ML models showed higher c-indexes with a range value of 0.92–0.94 comparing to conventional statistical methods in predicting biochemical recurrence after prostatectomy (21). Unfortunately, they considered a limited dataset without imputing the censored cases. Other sophisticated models based on active learning have been used to improve Cox regression and to predict prostate cancer survival among patients in the* Surveillance, Epidemiology, and End Results* (SEER) database, with c-indexes over 0.8 (22, 23).

We note that the use of the predictive models in predicting clinical outcomes (e.g., survival, grade, treatment, etc.) has become popular (24–26). However, to ensure a common understanding, data scientists and clinical researchers need to define a common set of outcome metrics. Defining ‘accuracy’ performance as the ratio of correct predictions to the total number of predictions is seldom appropriate in comparing predictive models, especially for survival analysis (27, 28). So far, the AUC and c-index, sensitivity and specificity, provide better performance metrics.

Whether deep learning performs better than conventional ML and statistical models in survival analysis remains unclear. The binary classification of tabular data is not the strength of neural network models (23). Recent breakthroughs based on deep learning (e.g., convolutional neural networks) and neural network algorithms rely primarily on deep analysis of medical images for a computer aided diagnosis (29, 30). Furthermore, the development of rigorous methods like neural networks to handle censored data with follow-up imaging may provide much better survival analyses for the future. Thus, the accuracy of our model is modest and could be enhanced by using a more contemporary approach such as MRI guided biopsies, with a central pathology review and a validation cohort.

The main asset of such models relies on their ability to be nurtured with prospectively acquired data, in order to gradually improve predictions. Moreover, a model could be shaped “locally” (learning from specific local databases) to take into account local specificities thus better be applicable to certain populations of patients. Nevertheless, there is still a need of prospective validation of these models before their integration from bench to bedside. Also, one of the downsides could potentially be related to the “black box” nature of algorithms such as DNN. Indeed, it is very difficult as an observer to decipher how the model intertwines the variables between them to eventually come with a prediction, possibly generating reluctancy among clinicians to use such tools. In daily practice, regarding the recent studies (31–33) published in the post-operative setting, such models can enhance the clinician decision making confidence for proposing adjuvant or salvage radiotherapy.

Our study has some limitations that should be noted. First, mpMRI data might be a promising addition dataset for improving the accuracy of the predictive models. The data analysis is represented by the fact that a standard ultra-sound guided prostate biopsy was used for most cases in this cohort. This does not reflect the current standard practice as MRI is now recommended in first line biopsy setting. Second, the median follow-up time was relatively short considering the natural history of the biochemical progression of intermediate-risk PCa. Third, the modifications to the Gleason score grading system in 2005 could have also introduced bias. In addition, the pathology data were not centralized among the different tertiary centers. However, only dedicated uropathologists reviewed the RP specimens at these referral centers, and to limit potential bias, we restricted our analyses to contemporary patients. Finally, we must admit that the difference found between AUC results is small.

## Conclusions

The results of this study indicate that predictive models could improve the prediction of 3-year BCR after RP based on routine variables used in CAPRA score with a population presenting intermediate-risk disease. Specifically, a deep neural network model showcased the highest performance metrics for predicting the BCR. This model will help clinicians to achieve the goal of personalized medicine and develop a strategic approach for prostate cancer treatment.

## Data Availability Statement

The original contributions presented in the study are included in the article/**Supplementary Material**. Further inquiries can be directed to the corresponding author.

## Author Contributions

Conceptualization, NL, PS, NG, J-BB. Methodology, NL, PS, and J-BB. Software, NL, NG. Validation, GG, MR, GP, FR, MS, RM, PM, TN, and VV-H. Formal analysis, NL, NG. Investigation, NL, PS, and J-BB. Resources, J-BB, MR, GP, FR, MS, RM, PM, TN, and VV-H. Data curation, PS, NL, and NG. Writing—original draft preparation, NL, NG, and PS. Writing—review and editing, NL, PS, NG, J-BB. Visualization, NL and J-BB. Supervision, PS and J-BB. Project administration, NL, PS, and J-BB. All authors contributed to the article and approved the submitted version

## Conflict of Interest

The authors declare that the research was conducted in the absence of any commercial or financial relationships that could be construed as a potential conflict of interest.

## References

[1] Wilt TJ Jones KM Barry MJ Andriole GL Culkin D Wheeler T Follow-up of prostatectomy versus observation for early prostate cancer. N Engl J Med (2017) 377:132–42. 10.1056/NEJMoa1615869 28700844

[2] Zumsteg ZS Spratt DE Pei I Zhang Z Yamada Y Kollmeier M A new risk classification system for therapeutic decision making with intermediate-risk prostate cancer patients undergoing dose-escalated external-beam radiation therapy. Eur Urol (2013) 64:895–902. 10.1016/j.eururo.2013.03.033 23541457

[3] Beauval J-B Cabarrou B Gandaglia G Patard P-M Ouzzane A de la Taille A External validation of a nomogram for identification of pathologically favorable disease in intermediate risk prostate cancer patients. Prostate (2017) 77:928–33. 10.1002/pros.23348 28370267

[4] Lubeck DP Litwin MS Henning JM Stier DM Mazonson P Fisk R The capsure database: a methodology for clinical practice and research in prostate cancer. Urology (1996) 48:773–7. 10.1016/S0090-4295(96)00226-9 8911524

[5] Cooperberg MR Pasta DJ Elkin EP Litwin MS Latini DM DuChane J The UCSF Cancer of the Prostate Risk Assessment (CAPRA) Score: a straightforward and reliable preoperative predictor of disease recurrence after radical prostatectomy. J Urol (2005) 173:1938–42. 10.1097/01.ju.0000158155.33890.e7 PMC2948569 15879786

[6] Cooperberg MR Hilton JF Carroll PR The CAPRA-S score: a straightforward tool for improved prediction of outcomes after radical prostatectomy. Cancer (2011) 117:5039–46. 10.1002/cncr.26169 PMC3170662 21647869

[7] Cooperberg MR Clinical risk-stratification for prostate cancer: where are we, and where do we need to go? Can Urol Assoc J (2017) 11:101–2. 10.5489/cuaj.4520 PMC5434505 28515808

[8] Breslow N Analysis of survival data under the proportional hazards model. Int Stat Rev Rev Int Stat (1975) 43:45–57. 10.2307/1402659

[9] Berkson J Application of the logistic function to bio-assay. J Am Stat Assoc (1944) 39:357–5. 10.2307/2280041

[10] Zupan B Demšar J Kattan MW Beck JR Bratko I Machine learning for survival analysis: a case study on recurrence of prostate cancer. Artif Intell Med (2000) 20:59–75. 10.1016/S0933-3657(00)00053-1 11185421

[11] Chaddad A Daniel P Sabri S Desrosiers C Abdulkarim B Integration of radiomic and multi-omic analyses predicts survival of newly diagnosed IDH1 wild-type glioblastoma. Cancers (2019) 11:1148. 10.3390/cancers11081148 PMC6721570 31405148

[12] Miotto R Wang F Wang S Jiang X Dudley JT Deep learning for healthcare: review, opportunities and challenges. Brief Bioinform (2018) 19:1236–46. 10.1093/bib/bbx044 PMC6455466 28481991

[13] Goldenberg SL Nir G Salcudean SE A new era: artificial intelligence and machine learning in prostate cancer. Nat Rev Urol (2019) 16:391. 10.1038/s41585-019-0193-3 31092914

[14] Ali O Shrestha A Soar J Wamba SF Cloud computing-enabled healthcare opportunities, issues, and applications: A systematic review. Int J Inf Manag (2018) 43:146–58. 10.1016/j.ijinfomgt.2018.07.009

[15] Prostate-specific antigen doubling time as a prognostic marker in prostate cancer. Eastham JA Nat Clin Pract Urol (2005) 2(10):482–91. 10.1038/ncpuro0321 16474622

[16] Hoffman RM Gilliland FD Eley JW Harlan LC Stephenson RA Stanford JL Racial and ethnic differences in advanced-stage prostate cancer: the prostate cancer outcomes study. J Natl Cancer Inst (2001) 93:388–95. 10.1093/jnci/93.5.388 11238701

[17] Cookson MS Aus G Burnett AL Canby-Hagino ED D’Amico AV Dmochowski R Variation in the definition of biochemical recurrence in patients treated for localized prostate cancer: the American Urological Association Prostate guidelines for localized prostate cancer update panel report and recommendations for a standard in the reporting of surgical outcomes. J Urol (2007) 177:540–5. 10.1016/S0084-4071(08)70132-5 17222629

[18] Abadi M Barham P Chen J Chen Z Davis A Dean J A system for large-scale machine learning. Proc 12th USENIX Symp Oper Syst Des Implementation OSDI 2016 (2016). p. 265–83 Available at: https://www.usenix.org/system/files/conference/osdi16/osdi16-abadi.pdf.

[19] Jambor I Falagario U Ratnani P Perez IM Demir K Merisaari H Prediction of biochemical recurrence in prostate cancer patients who underwent prostatectomy using routine clinical prostate multiparametric MRI and decipher genomic score. J Magn Reson Imaging (2020) 51:1075–85. 10.1002/jmri.26928 31566845

[20] Brajtbord JS Leapman MS Cooperberg MR The CAPRA score at 10 years: contemporary perspectives and analysis of supporting studies. Eur Urol (2017) 71:705–9. 10.1016/j.eururo.2016.08.065 27616723

[21] Wong NC Lam C Patterson L Shayegan B Use of machine learning to predict early biochemical recurrence after robot-assisted prostatectomy. BJU Int (2019) 123:51–7. 10.1111/bju.14477 29969172

[22] Wen H Li S Li W Li J Yin C Comparison of four machine learning techniques for the prediction of prostate cancer survivability. In: . 15th Int Comput Conf Wavelet Act Media Technol Inf Process ICCWAMTIP. Chengdu, China: IEEE (2018). p. 112–6 Available at: https://ieeexplore.ieee.org/document/8632577/.

[23] Nezhad MZ Sadati N Yang K Zhu D A deep active survival analysis approach for precision treatment recommendations: application of prostate cancer. Expert Syst Appl (2019) 115:16–26. 10.1016/j.eswa.2018.07.070

[24] Ajay K Sushil R Tiwari A Cancer survival analysis using machine learning. SSRN Electron J (2019) 115:16–26. 10.2139/ssrn.3354469

[25] Chaddad A Niazi T Probst S Bladou F Anidjar M Bahoric B Predicting Gleason score of prostate cancer patients using radiomic analysis. Front Oncol (2018) 8:630. 10.3389/fonc.2018.00630 30619764 PMC6305278

[26] Chaddad A Kucharczyk MJ Niazi T Multimodal radiomic features for the predicting Gleason score of prostate cancer. Cancers (2018) 10:249. 10.3390/cancers10080249 PMC6116195 30060575

[27] Huang J Ling CX Using AUC and accuracy in evaluating learning algorithms. IEEE Trans Knowl Data Eng (2005) 17:299–310. 10.1109/TKDE.2005.50

[28] Ling CX Huang J Zhang H AUC: a better measure than accuracy in comparing learning algorithms. In: Xiang Y Chaib-Draa B, editors. Adv Artif Intell. Springer Berlin Heidelberg (2003). p. 329–41.

[29] Liu S Zheng H Feng Y Li W Prostate cancer diagnosis using deep learning with 3D multiparametric MRI. Med Imaging 2017 Comput-Aided Diagn. Int Soc Optics Photonics (2017) 10134:1013428. 10.1117/12.2277121

[30] Le MH Chen J Wang L Wang Z Liu W Cheng KT Automated diagnosis of prostate cancer in multi-parametric MRI based on multimodal convolutional neural networks. Phys Med Biol (2017) 62:6497–514. 10.1088/1361-6560/aa7731 28582269

[31] Parker CC Clarke NW Cook AD Kynaston HG Petersen PM Catton C Timing of radiotherapy after radical prostatectomy (RADICALS-RT): a randomised, controlled phase 3 trial. Lancet (2020) 396:1413–21. 10.1016/S0140-6736(20)31553-1 PMC7616947 33002429

[32] Vale CL Fisher D Kneebone A Parker C Pearse M Richaud P Adjuvant or early salvage radiotherapy for the treatment of localised and locally advanced prostate cancer: a prospectively planned systematic review and meta-analysis of aggregate data. Lancet (2020) 396:1422–31. 10.1016/S0140-6736(20)31952-8 PMC7611137 33002431

[33] Sargos P Chabaud S Latorzeff I Magné N Benyoucef A Supiot S Adjuvant radiotherapy versus early salvage radiotherapy plus short-term androgen deprivation therapy in men with localised prostate cancer after radical prostatectomy (GETUG-AFU 17): a randomised, phase 3 trial. Lancet Oncol (2020) 21:1341–52. 10.1016/S1470-2045(20)30454-X 33002438

