# Supplementary material for: Deep Neural Networks Outperform the CAPRA Score in Predicting Biochemical Recurrence After Prostatectomy
Source: Front Oncol. 2021 Feb 11;10:607923. doi: 10.3389/fonc.2020.607923 (PMC7906005; doi:10.3389/fonc.2020.607923)
Supplement: Supplementary file 1 [file DataSheet_1.docx]

**Supplementary material. Tuned predictive models**

**For each hyperparameter,** the range and step used in the grid search, over numeric parameters, asrecommended in the scikit/sklearn and Keras documentation, are presented in the columns. Unmentioned parameters remain at their respective default values. The hyperparameter’s values showing the highest AUC are also reported in the last column.

| **Hyperparameter** | **Tested range (step)** | **Value with the highest AUC** |
| --- | --- | --- |
| **Logistic Regression** | | |
| Solver | liblinear | liblinear |
| C-value | 0.1-5 (0.1) | 3.0 |
| **K-neighbor** | | |
|  |  |  |
| Number of neighbors | 3-20 (1) | 5 |
| Leaf size | 20-50 (10) | 30 |
| **Random Forest** | | |
| Number of estimators | 10-500 (10) | 220 |
| Maximal features | 2-10 (1) | 6 |
| **Dense Neural Networks** | | |
| Number of hidden layers | 1-5 (1) | 3 |
| Number of nodes  in each layer | 32-128 (32) | 64 |
| Kernel type | normal / random uniform | normal |
| Learning rate | 0.00001-0.1 (0.0005) | 0.008 |
| Shuffling of input samples | Yes | Yes |
| Batch size for input layer | 1-256 (32) |  |
| Activation function  for last layer | Sigmoid | Sigmoid |
| Activation function type  for other layers | ReLu / Sigmoid / Tanh | Sigmoid |
| Loss function | Binary cross-entropy | Binary cross-entropy |
| Optimizer | Adam / SGD | Adam |
